# Development and validation of short-term renal prognosis prediction model in diabetic patients with acute kidney injury

**DOI:** 10.1186/s13098-022-00971-1

**Published:** 2022-12-27

**Authors:** Manqiu Mo, Zichun Huang, Tianyun Gao, Yuzhen Luo, Xiaojie Pan, Zhenhua Yang, Ning Xia, Yunhua Liao, Ling Pan

**Affiliations:** 1 Geriatric Department of Endocrinology, The First Affiliated Hospital of Guangxi Medical University, Nanning, 530021 China; 2 Department of Cardiovascular Thoracic Surgery, Nanning Second People’s Hospital, The Third Affiliated Hospital of Guangxi Medical University, Nanning, 530031 China; 3 Department of Nephrology, The First Affiliated Hospital of Guangxi Medical University, Nanning, 530021 China

**Keywords:** Diabetes, Acute kidney injury, Renal function, Prediction model

## Abstract

**Objective:**

Diabetes is a major cause of the progression of acute kidney injury (AKI). Few prediction models have been developed to predict the renal prognosis in diabetic patients with AKI so far. The aim of this study was to develop and validate a predictive model to identify high-risk individuals with non-recovery of renal function at 90 days in diabetic patients with AKI.

**Methods:**

Demographic data and related laboratory indicators of diabetic patients with AKI in the First Affiliated Hospital of Guangxi Medical University from January 31, 2012 to January 31, 2022 were retrospectively analysed, and patients were followed up to 90 days after AKI diagnosis. Based on the results of Logistic regression, a model predicting the risk of non-recovery of renal function at 90 days in diabetic patients with AKI was developed and internal validated. Consistency index (C-index), calibration curve, and decision curve analysis were used to evaluate the differentiation, accuracy, and clinical utility of the prediction model, respectively.

**Results:**

A total of 916 diabetic patients with AKI were enrolled, with a male to female ratio of 2.14:1. The rate of non-recovery of renal function at 90 days was 66.8% (612/916). There were 641 in development cohort and 275 in validation cohort (ration of 7:3). In the development cohort, a prediction model was developed based on the results of Logistic regression analysis. The variables included in the model were: diabetes duration (OR = 1.022, 95% CI  1.012–1.032), hypertension (OR = 1.574, 95% CI 1.043–2.377), chronic kidney disease (OR = 2.241, 95% CI 1.399–3.591), platelet (OR = 0.997, 95% CI 0.995–1.000), 25-hydroxyvitamin D3 (OR = 0.966, 95% CI  0.956–0.976), postprandial blood glucose (OR = 1.104, 95% CI 1.032–1.181), discharged serum creatinine (OR = 1.003, 95% CI 1.001–1.005). The C-indices of the prediction model were 0.807 (95% CI 0.738–0.875) and 0.803 (95% CI 0.713–0.893) in the development and validation cohorts, respectively. The calibration curves were all close to the straight line with slope 1. The decision curve analysis showed that in a wide range of threshold probabilities.

**Conclusion:**

A prediction model was developed to help predict short-term renal prognosis of diabetic patients with AKI, which has been verified to have good differentiation, calibration degree and clinical practicability.

## Background

Due to overweight, obesity and unhealthy lifestyles, the global prevalence of diabetes is increasing year by year. There are currently about 537 million adults worldwide with diabetes, and the number is expected to rise to 643 million by 2030 and 783 million by 2045 [1, 2]. Diabetes, as a highly prevalent disease with complications affecting multiple systems of the human body, has become an important burden affecting global health. The kidney is one of the important target organs of diabetic vascular damage. It is estimated that more than 60% of patients with diabetes in Asia will develop renal complications [3]. Therefore, it is very important for diabetic patients to recognize and control their renal complications.

Acute kidney injury (AKI) has been recognized as one of the common renal complications of diabetes [4]. Diabetes patients have high prevalence of AKI, and diabetic patients with AKI have worse prognosis than those without diabetes [5]. Diabetes can not only further increase the risk of AKI progress to chronic renal failure (CRF) or end stage renal disease (ESRD), but also increase the risk of mortality, cardiovascular complication and other adverse consequences [6–8]. A retrospective cohort showed that the prevalence of AKI in diabetic patients was as high as 48.6%, and diabetic patients were more likely to have chronic kidney disease (CKD) and CRF than non-diabetic patients (46.3% vs. 17.2%) [9]. Data from the Centers for Disease Control and Prevention also indicate that diabetes is present in up to 40% of all hospitalized patients with AKI [10]. In addition, diabetes complicated with AKI not only increases the length of hospitalization and treatment expenditure of patients, but also seriously wastes social and medical resources [11]. Therefore, there is an urgent need for a simple and easy method to rapidly assess the risk of non-recovery of renal function in diabetic patients with AKI.

A nomogram is a user-friendly tool with a graphical representation that can be used to calculate the probability of a specific event for each individual [12, 13]. Early prediction, prevention and treatment of AKI should be benefit for improving the prognosis of diabetes. In addition, a prospective cohort study showed that non-recovery of renal function at 90 days after AKI was an independent risk factor for progression of CKD (OR = 28.03, 95% CI 5.37–146.31) [14]. However, there are few studies on short-term renal outcomes in diabetic patients with AKI, and risk prediction tools are lacking to identify high-risk individuals with non-recovery of renal function at 90 days. This study aims to provide a practical risk stratification method for early prediction of adverse renal outcomes in diabetic patients with AKI.

## Materials and methods

### Research subjects

Patients diagnosed with diabetes and AKI in the First Affiliated Hospital of Guangxi Medical University from January 31, 2012 to January 31, 2022 were selected as the research subjects. Inclusion criteria: (1) diabetes was diagnosed before AKI; (2) changes in serum creatinine (Scr) consistent with the diagnostic criteria for AKI; (3) the follow-up time was no less than 90 days. Exclusion criteria: (1) age < 18 years; (2) CKD stage 5 or renal replacement therapy (RRT); (3) death or loss to follow-up within 90 days of AKI diagnosis; (4) patients with missing important baseline data and the Scr values of 90-day following up. This study was approved by the Ethics Committee of the First Affiliated Hospital of Guangxi Medical University [Approval number: 2019(KY-E-028)]. Participants were required to sign (or have a proxy sign) informed consent for data collection. This study was conducted in accordance with the tenets of the Declaration of Helsinki.

### Research groupings

Using a retrospective cohort study, the patients were followed up from the diagnosis of AKI to 90 days. The observed clinical outcome was the recovery of renal function. All subjects were randomly divided into training cohort and validation cohorts by a ratio of 7:3. The development cohort was used to construct the prediction model, and validation cohort was used to verify the model’s prediction ability to predict non-recovery of renal function at 90 days in diabetic patients with AKI.

### Clinical data collection

Demographic data and baseline clinical data were collected, including age, gender, duration of diabetes (months), underlying diseases (hypertension, CKD), body mass index (BMI), blood pressure, blood routine, liver function, renal function, electrolytes, myocardial enzymes, 25-hydroxyvitamin D3 [25(OH)D3], blood glucose, and discharged Scr, dialysis, etc. The baseline Scr was defined as a stable Scr within the last 3 months or longer if none was available within 3 months [15]. The discharged Scr was the last inpatient Scr value measured before AKI hospital discharge [16]. Other laboratory indicators were the first time of values after hospitalization.

### Data preprocessing

Several steps were used in data preprocessing. Before starting the model building process, we have checked each variable for missing, outliers, or implausible values. As only a small number of missing values were present (< 5% of each variable), the missing data and outliers were removed.

### Diagnostic criteria

AKI was defined referring to the diagnostic criteria of AKI in the Kidney Disease: Improving Global Outcomes guideline: Scr increased by ≥ 26.5 µmol/L within 48 h or increased by > 50% of the baseline value within 7 days. The criteria of AKI stage: stage 1: Scr increase to 1.5–1.9 times of baseline value or increased ≥ 0.3 mg/dL, stage 2: Scr increase to 2.0–2.9 times of base value, stage 3: Scr increase to 3 times or ≥ 4.0 mg/ dL or begin RRT [17].

Diagnosis of diabetes conforms to World Health Organization criteria as follows: (1) random blood glucose ≥ 11.1 mmol/L, (2) fasting blood glucose (FBG) ≥ 7.0 mmol/L, or (3) postprandial blood glucose (PBG) ≥ 11.1 mmol/L [18].

Renal function recovery was defined as a return of Scr to less than 1.25 times the baseline value or Scr decrease to normal lab range (Scr ≤ 104 µmol/L in males or Scr ≤ 84 µmol/L in females) or removal of RRT [19]. Otherwise, it is considered to be non-recovery of renal function.

### Statistical analysis

All statistical analyses were performed on SPSS 22.0 and R 3.6.3 software. For continuous variables, data are presented as the mean ± standard deviation; for dichotomous variables, data are presented as whole numbers and proportions [n(%)]. Comparisons among two groups were performed by χ^2^ test. The univariate Logistic regression model was used to screen the risk factors, the variables with *P* < 0.05 were included in the multivariate Logistic regression (the forward LR method). Based on the multivariate Logistic analysis results, a nomogram was constructed based on the odds ratios of risk factors. The concordance index (C-index) and receiver operating characteristic curve (ROC) were used to evaluate the discrimination of the prediction model in the development and validation cohorts. The Hosmer-Lemeshow goodness of fit test was used for the calibration degree to compare the difference between the predicted probability and the actual probability, and *P* > 0.05 indicated that the model calibration degree was reliable. The Brier score was also calculated to evaluate the calibration degree of the model, and the better the calibration of the model with a Brier score close to 0. The Bootstrap method was used to draw the calibration curve, and the closer the slope is to 1, the higher the accuracy of the prediction model. Decision curve analysis was conducted to determine the clinical usefulness of the nomogram by quantifying the net benefit at different threshold probabilities in the primary dataset. *P* < 0.05 was regarded as a statistically significant difference.

## Results

### Characteristics of patients in the development and validation cohorts

Table 1 shows the characteristics of the patients in the development and validation cohorts. Among 1280 diabetic patients with AKI, 52 were younger than 18 years old, 98 had stage 5 CKD or had regular RRT, 36 had incomplete baseline data, 152 died during follow-up, and 26 were lost to follow-up. Finally, a total of 916 patients were included in this study, with an average age of 61.31 ± 13.4 years and a male to female ratio of 2.14:1. About 39.7% of the patients had history of CKD (364/916); 402 patients (43.9%) with stage 1 AKI, 337 patients (36.8%) with stage 2 AKI, and 177 cases (19.3%) in stage 3 AKI; the 90-day non-recovery rate of renal function was 66.8% (612/916). Diabetic patients with AKI were randomly divided into 641 cases in the development cohort and 275 cases in the validation cohort. The 90-day renal function recovery rate in the development cohort was 34.9% (224/641), and the 90-day renal function recovery rate in the validation cohort was 29.1% (80/275).

**Table 1:** The difference in development cohort and the validation cohort in terms of the demographic characteristics, laboratory values

Parameters	Total	Development cohort	Validation cohort	t/χ^2^	*P*-value
Male/female	624/292	435/206	189/86	0.066	0.797
Age (year)	61.31 ± 13.40	61.13 ± 13.21	61.75 ± 13.85	− 0.642	0.521
Length of stay (day)	25.66 ± 51.77	20.39 ± 16.97	21.39 ± 18.21	− 0.723	0.470
Diabetes duration (month)	43.18 ± 24.77	44.78 ± 26.14	39.98 ± 21.48	2.920	0.004
Hypertension [n(%)]	566 (61.8)	393 (61.3)	173 (62.9)	0.208	0.648
CKD [n(%)]	364 (39.7)	258 (40.2)	106 (38.5)	0.233	0.629
BMI (kg/m^2^)	24.04 ± 3.71	23.80 ± 3.86	24.16 ± 4.24	− 1.063	0.288
PP (mmHg)	58.62 ± 18.38	57.83 ± 18.38	59.51 ± 17.70	− 1.273	0.203
MAP (mmHg)	95.79 ± 17.24	95.64 ± 18.17	96.90 ± 17.05	− 0.971	0.332
WBC (×10^9^/L)	12.51 ± 7.68	12.63 ± 8.13	12.21 ± 6.52	0.782	0.434
Hb (g/L)	104.06 ± 23.28	104.56 ± 23.11	102.90 ± 23.67	0.934	0.350
PLT (×10^9^/L)	191.55 ± 86.90	189.51 ± 82.64	196.32 ± 96.15	− 1.024	0.306
Alb (g/L)	32.27 ± 7.16	32.20 ± 7.94	31.96 ± 7.71	0.393	0.695
25(OH)D3 (nmol/L)	57.23 ± 23.29	57.36 ± 23.65	56.92 ± 22.47	0.265	0.791
NT-proBNP (pg/mL)	3632.98 ± 1851.39	4122.55 ± 1701.35	4017.90 ± 1422.01	0.880	0.379
FBG (mmol/L)	8.39 ± 2.94	8.46 ± 3.49	8.22 ± 3.42	0.834	0.405
PBG (mmol/L)	12.44 ± 2.80	12.41 ± 3.95	12.34 ± 4.15	0.170	0.865
HbA1c (%)	7.52 ± 1.36	7.49 ± 1.62	7.48 ± 1.74	0.102	0.918
BUN (mmol/L)	13.89 ± 8.85	14.00 ± 9.97	14.15 ± 10.28	− 0.194	0.846
Baseline Scr (µmol/L)	132.53 ± 102.00	141.09 ± 116.94	149.80 ± 139.41	− 0.798	0.425
UA (µmol/L)	422.60 ± 196.55	418.43 ± 197.07	410.48 ± 203.23	0.540	0.589
HCO3^−^ (mmol/L)	22.04 ± 5.23	22.13 ± 5.18	21.82 ± 5.36	0.807	0.420
Cystatin C (mg/L)	2.31 ± 1.45	2.35 ± 1.49	2.24 ± 1.36	1.011	0.312
Serum potassium (mmol/L)	4.19 ± 0.81	4.20 ± 0.81	4.17 ± 0.81	0.492	0.623
AKI stage				3.074	0.215
1	402 (43.9)	273 (42.6)	129 (46.9)		
2	337 (36.8)	235 (36.7)	102 (37.1)		
3	177 (19.3)	133 (20.7)	44 (16.0)		
Discharged Scr (µmol/L)	215.17 ± 160.55	217.02 ± 160.71	210.88 ± 160.38	0.530	0.596
RRT [n(%)]	186 (20.3)	140 (21.8)	46 (16.7)	3.110	0.078
Renal recovery	304 (33.2)	224 (34.9)	80 (29.1)	2.975	0.085

*25(OH)D3* 25-hydroxyvitamin D3; *AKI* acute kidney injury; *Alb* albumin; *BMI* body mass index; *BUN* blood urea nitrogen; *CKD* chronic kidney disease; *FBG* fasting blood glucose; *Hb* hemoglobin; *HbA1c* hemoglobin A1c; *MAP* mean arterial pressure; *NT-proBNP* N-terminal prohormone of brain natriuretic peptide; *PBG* postprandial blood glucose; *PLT* platelet count; *PP* pulse pressure; *RRT* renal replacement therapy; *Scr* Serum creatinine; *UA* uric acid; *WBC* white blood cell count

Compared with the validation cohort, the development cohort had a longer diabetes duration (p < 0.05). There was no significant difference in gender, age, length of hospital stay, history of hypertension, CKD, BMI, blood pressure, white blood cell count (WBC), hemoglobin, platelet (PLT), albumin, 25(OH)D3, N-terminal prohormone of brain natriuretic peptide (NT-proBNP), FBG, PBG, glycated hemoglobin A1c (HbA1c), blood urea nitrogen (BUN), baseline Scr, uric acid (UA), serum potassium level, AKI stage, RRT rate, discharge Scr and renal function non-recovery rate in development and validation cohorts.

### Risk factors affecting 90-day renal outcomes

Logistic regression analysis was used to construct the prediction model, as shown in Table 2. Univariate logistic regression analysis of the development cohort showed that, the diabetes duration, history of hypertension, CKD, mean arterial pressure, WBC, PLT, 25(OH)D3, NT-proBNP, serum potassium, PBG, discharge Scr, and RRT rate were risk factors that affect the renal function of diabetic patients with AKI (p < 0.05). The above variables were included in logistic regression analysis. The results showed that increase of diabetes duration (OR = 1.022, 95% CI 1.012–1.032), history of hypertension (OR = 1.574, 95% CI 1.043–2.377), history of CKD (OR = 2.241, 95% CI 1.399–3.591), decrease of PLT (OR = 0.997, 95% CI 0.995–1.000), lower 25(OH)D3 (OR = 0.966, 95% CI 0.956–0.976), increase of PBG (OR = 1.104, 95% CI 1.032–1.181), increase of discharged Scr (OR = 1.003, 95% CI 1.001–1.005) are the independent risk factors affecting the 90-day non-recovery of function of diabetic patients with AKI (as shown in Table 3).

**Table 2:** Univariate logistic regression analysis of factors relating to adverse renal outcome in the development cohort

Parameters	B	S.E.	Wald	OR (95%CI)	*P*-value
Male	0.157	0.176	0.790	1.169 (0.828–1.652)	0.374
Age (year)	0.007	0.006	1.307	1.007 (0.995–1.019)	0.253
Length of stay (day)	0	0.002	0.045	1.000 (0.997–1.003)	0.831
Diabetes duration (month)	0.010	0.004	7.620	1.010 (1.003–1.017)	0.006
Hypertension [n(%)]	0.497	0.169	8.635	1.643 (1.180–2.288)	0.003
CKD [n(%)]	1.387	0.192	52.040	4.002 (2.745–5.833)	< 0.001
BMI (kg/m^2^)	0.042	0.023	3.350	1.042 (0.997–1.090)	0.067
PP (mmHg)	0.005	0.004	1.042	1.005 (0.996–1.013)	0.307
MAP (mmHg)	0.011	0.005	4.960	1.011 (1.001–1.021)	0.026
WBC (×10^9^/L)	0.024	0.012	4.011	1.025 (1.001–1.049)	0.045
Hb (g/L)	− 0.001	0.004	0.123	0.999 (0.991–1.006)	0.726
PLT (×10^9^/L)	− 0.003	0.001	7.788	0.997 (0.995–0.999)	0.005
Alb (g/L)	− 0.009	0.012	0.651	0.991 (0.968–1.014)	0.420
25(OH)D3	− 0.047	0.005	97.124	0.954 (0.945–0.963)	< 0.001
NT-proBNP (pg/mL)	0.002	0	146.514	1.002 (1.002–1.002)	< 0.001
FBG (mmol/L)	0.021	0.029	0.550	1.022 (0.965–1.081)	0.458
PBG (mmol/L)	0.136	0.032	17.903	1.146 (1.076–1.221)	< 0.001
HbA1c (%)	− 0.058	0.060	0.917	0.944 (0.839–1.062)	0.338
BUN (mmol/L)	− 0.008	0.010	0.658	0.992 (0.974–1.011)	0.417
Baseline Scr (µmol/L)	0	0.001	0.217	1.000 (0.998–1.001)	0.641
UA (µmol/L)	0	0	0.552	1.000 (0.999–1.001)	0.457
HCO3- (mmol/L)	− 0.031	0.017	3.451	0.969 (0.938–1.002)	0.063
Cystatin C (mg/L)	0.071	0.060	1.414	1.074 (0.955–1.208)	0.234
Serum potassium (mmol/L)	0.301	0.117	6.604	1.351 (1.074–1.699)	0.010
AKI stage			1.947		0.378
1				1 [Reference]	
2	0.245	0.188	1.693	1.278 (0.883–1.848)	0.193
3	0.007	0.219	0.001	1.007 (0.655–1.547)	0.976
Discharged Scr (µmol/L)	0.005	0.001	38.774	1.005 (1.003–1.006)	< 0.001
RRT [n(%)]	0.551	0.214	6.616	1.735 (1.140–2.641)	0.010

*25(OH)D3* 25-hydroxyvitamin D3; *AKI* acute kidney injury; *Alb* albumin; *BMI* body mass index; *BUN* blood urea nitrogen; *CKD* chronic kidney disease; *FBG* fasting blood glucose; *Hb* hemoglobin; *HbA1c* hemoglobin A1c; *MAP* mean arterial pressure; *NT-proBNP* N-terminal prohormone of brain natriuretic peptide; *PBG* postprandial blood glucose; *PLT* platelet count; *PP* pulse pressure; *RRT* renal replacement therapy; *Scr* Serum creatinine; *UA* uric acid; WBC white blood cell count

**Table 3:** Multivariate logistic regression analysis of factors relating to adverse renal outcome in the development cohort

Parameters	B	S.E.	Wald	OR (95%CI)	*P*-value
Diabetes duration	0.022	0.005	19.462	1.022 (1.012–1.032)	< 0.001
Hypertension	0.454	0.210	4.660	1.574 (1.043–2.377)	0.031
CKD	0.807	0.241	11.258	2.241 (1.399–3.591)	0.001
PLT	− 0.003	0.001	5.188	0.997 (0.995-1.000)	0.023
25(OH)D3	− 0.034	0.005	42.164	0.966 (0.956–0.976)	< 0.001
PBG	0.099	0.034	8.367	1.104 (1.032–1.181)	0.004
Discharged Scr	0.003	0.001	9.228	1.003 (1.001–1.005)	0.002

*25(OH)D3* 25-hydroxyvitamin D3; *CKD* chronic kidney disease; *PBG* postprandial blood glucose; *PLT* platelet count; *Scr* Serum creatinine

### Construction and validation of predictive models

**Fig. 1:**
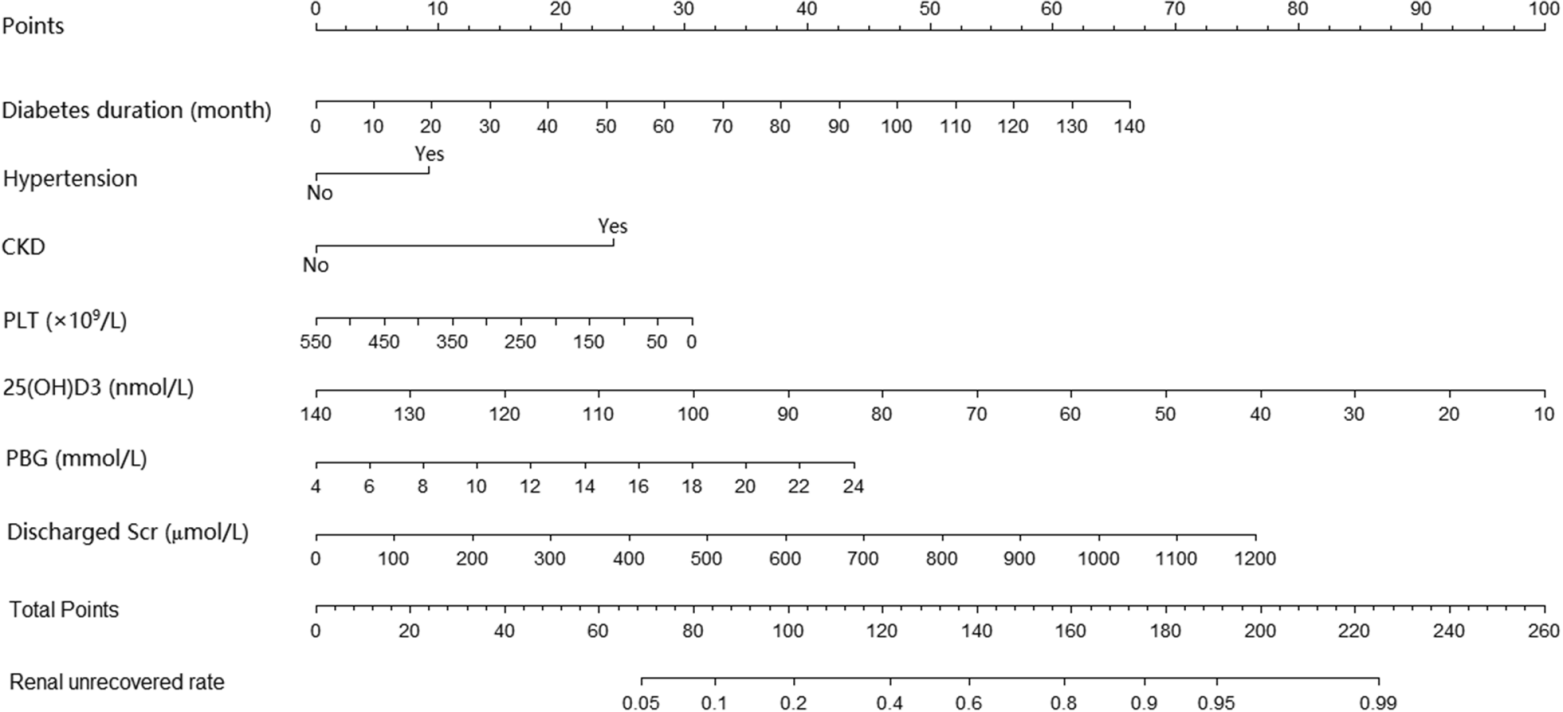
Nomogram predicting the 90-day renal unrecovered rate in diabetic patients with acute kidney injury (AKI).

Based on the above results, a predictive model for 90-day renal prognosis risk in diabetic patients with AKI was constructed. The nomogram is shown in Fig. 1. By calculating the scores corresponding to the risk factors, the individual’s non-recovery rate of renal function at 90 days after the diagnosis of AKI was estimated. Taking a patient as an example, when predicting the 90-day non-recovery rate of renal function, the score of each influencing factor is calculated first. For example, 14 points for the diabetes duration of 30 months, 10 points for history of hypertension, 0 point for having no history of CKD, 22.5 points for PLT of 150 × 10^9^/L, 68.75 points for 25(OH)D3 of 50 nmol/L, 18.75 points for PBG of 12 mmol/L, 20 points for discharge Scr of 300 µmol/L. The total score of this patient was 154 points (14 + 10 + 0 + 22.5 + 68.75 + 18.75 + 20), the risk of non-recovery rate of renal function at 90 days in diabetic patients with AKI was about 72.5%.

**Fig. 2:**
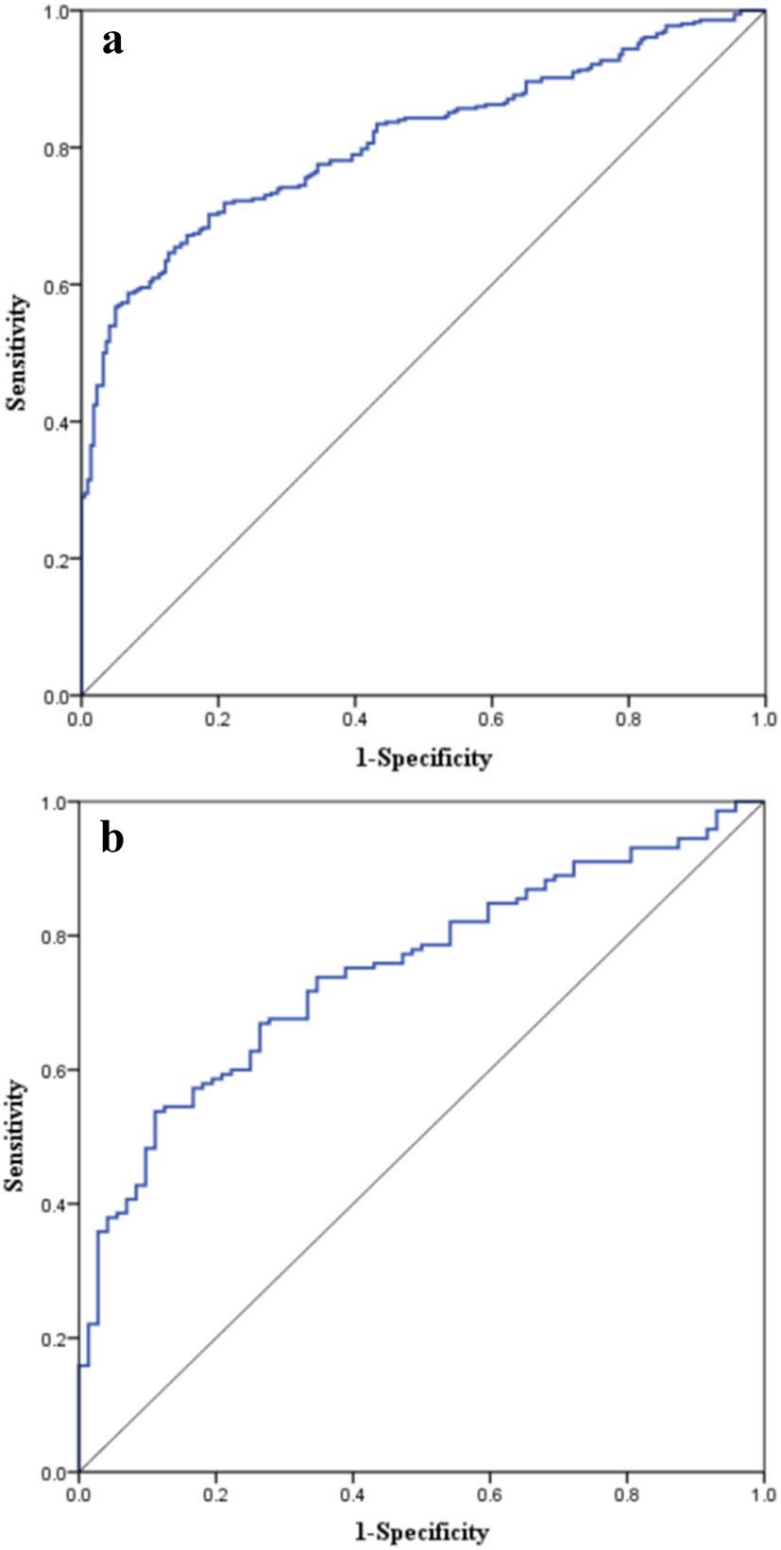
**A** Receiver operating characteristic (ROC) curve of the prediction model in the development cohort. **B** ROC curve of the prediction model in the validation cohort

**Fig. 3:**
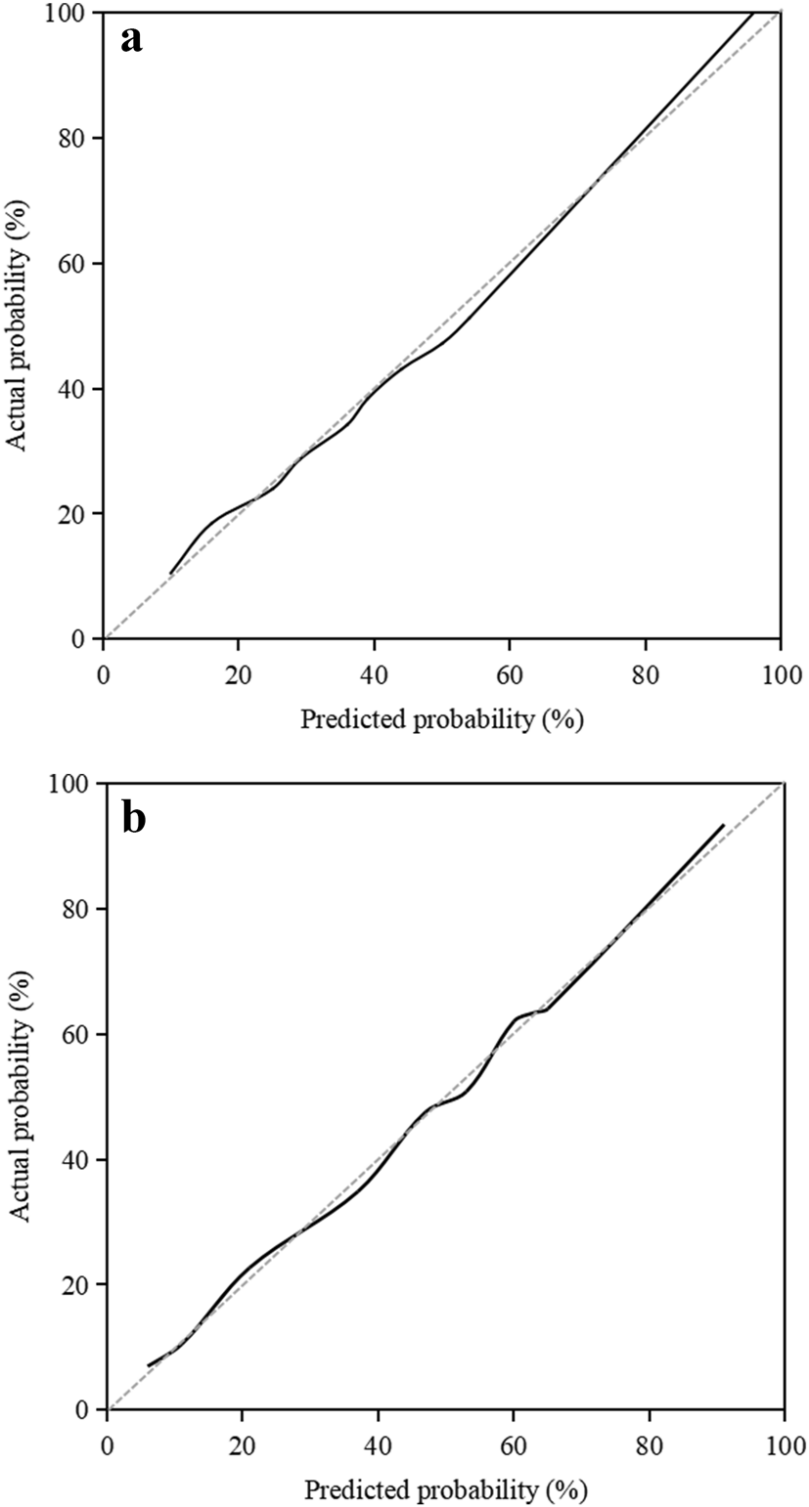
**A** Calibration curve of the nomogram in the development cohort. **B** Calibration curve of the nomogram in the validation cohort

The C-index of the prediction model in the development cohort was 0.807 (95% CI  0.738–0.875), the area under the ROC curve (AUROC) of the prediction model for predicting 90-day non-recovered renal function in diabetic patients with AKI was 0.809, and the Youden index was 0.619, the sensitivity and specificity were 0.746 and 0.873, respectively (Fig. 2A). The Hosmer-Lemeshow test of the model in the development cohort was *P* = 0.326, the Brier score was 0.065, and the calibration curve was a straight line with a slope close to 1 (Fig. 3A). The C-index of the prediction model in the validation cohort was 0.803 (95% CI 0.713–0.893). As shown in Fig. 2B, the AUROC for predicting the 90-day non-recovered renal function in diabetic patients with AKI was 0.748, and the Youden index was 0.598, the sensitivity and specificity were 0.709 and 0.889, respectively. The Hosmer-Lemeshow test of the model in the validation cohort was *P* = 0.117, the Brier score was 0.082, and the calibration curves were all close to a straight line with a slope of 1 (Fig. 3B).

### Clinical use of the model

**Fig. 4:**
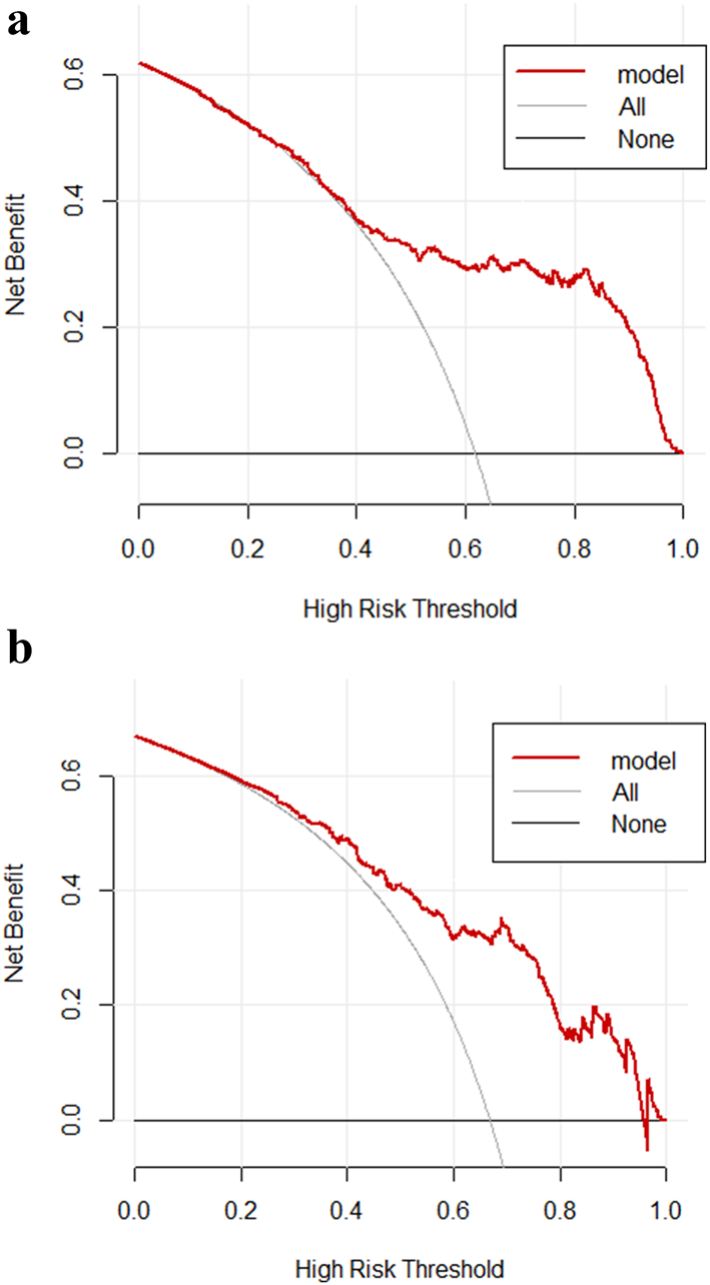
**A** Decision curve analysis for the nomogram in the development cohort. **B** Decision curve analysis for the nomogram in the validation cohort

Decision curve analysis demonstrated the clinical utility of the prediction model based on a continuous potential risk threshold (x-axis) and the net benefit of using the model to risk stratify patients (y-axis). The black line represents that all patients have recovery of renal function, and the gray line represents that all patients have non-recovery of renal function. The threshold probability of the predictive model in the development cohort was 25–100% to produce the greatest benefit (Fig. 4A), and the threshold probability of the predictive model in the validation cohort was in the range of 20–96% to yield the greatest benefit (Fig. 4B), indicating that the model has good clinical practicability in predicting the 90-day non-recovery of renal function in diabetic patients with AKI. For example, if diabetic patients with AKI has an individual threshold probability of 40% (i.e., if he has a > 40% risk of non-recovery of renal function at 90 days, he will choose to undergo further detailed screening), use the nomogram to decide whether to proceed on further detailed screening, its net benefit was 0.35 in the development cohort (0.50 in the validation cohort).

## Discussion

In this study, the influencing factors of short-term renal prognosis were evaluated based on the demographic characteristics and clinical data of diabetic patients with AKI, and a prediction model of prognostic risk was constructed. The results showed that diabetes duration, history of hypertension and CKD, increase of PBG and discharged Scr were the risk factors for non-recovery of renal function at 90-day in diabetic patients with AKI; while decrease of PLT and 25(OH)D3 were the protective factors. The prediction model has good discrimination and accuracy through internal verification of Bootstrapping resampling techniques with 1,000 replications and validation cohort. The decision curve analysis also showed good clinical utility. In addition, the predictors used in this model are readily available at discharge and can be used to predict short-term renal prognosis in diabetic patients with AKI, and patients are individually assessed and followed up using this risk stratification [20]. Moreover, early intervention in high-risk patients who had non-recovery of renal function at 90 days may improve renal outcomes and survival in diabetic patients with AKI.

Numerous AKI prediction models have been reported in the previous literature. However, most of prognostic models were developed for specific patient populations (e.g., septic shock, post-cardiac surgery, or critically ill patients) [21–23]. Previous study by our team had constructed a prediction model for all-cause mortality in diabetic patients with AKI based on advanced age, low pulse pressure difference, low 25(OH)D3 and multiple organ dysfunction syndrome, and the C-indices were 0.880 and 0.798 in development and validation cohorts, respectively [24]. Diabetes increases the risk of poor renal prognosis in patients with AKI. Then, the clinical question arises: what are the risk factors of poor renal prognosis for diabetic patients with AKI? At present, there are still few predictive models for the non-recovery of renal function in diabetic patients with AKI. In this study, based on the multivariate Logistic stepwise regression analysis results, a predictive model for 90-day non-recovery of renal function in diabetic patients with AKI was constructed. The final variables included in the model were: duration of diabetes, history of CKD, history of hypertension, PLT, 25(OH)D3, PBG, discharged Scr. We hoped that this prediction model could be used to guide clinicians in their decisions to aid prognostication or to stratify disease severity and treatment.

A prospective study showed that, compared with patients with diabetes duration of less than 5 years, patients with diabetes duration of more than 20 years had a faster decline in renal function [25]. Patients with diabetes with a longer duration generally had poorer glycemic control and were more likely to have other comorbidities. Moreover, metabolic disease, and microvascular disease may be more severe [26]. These adverse risk factors may make it more difficult to recovery of renal function in diabetic patients with AKI, which is consistent with the results of high diabetes duration and PBG in our prediction model. History of CKD and hypertension are traditional risk factors for AKI in diabetic patients, as well as risk factors for the progression of AKI to CKD and ESRD [27, 28]. An observational study showed that critically ill patients with a history of CKD to admission had a significantly increased risk of death [29]. Patients with AKI in addition to CKD are at very high risk for ESRD [27]. Diabetic patients with 25(OH)D < 20 ng/ml and 25(OH)D < 30 ng/ml had a higher risk of developing proteinuria (OR = 2.8, 95% CI 1.6–4.9; OR = 2.1, 95% CI 1.3–4.6) [30]. Vitamin D may reduce oxidative stress by enhancing renal antioxidant capacity [31, 32], prevent damage to podocytes by inhibiting hyperglycemia-induced apoptosis, promote anti-inflammatory effects, and improve endothelial function, thereby exerting renal protection [33]. These findings were also confirmed by our regression model, with low 25(OH)D being an independent risk factor for non-recovery of renal function in diabetic patients with AKI. A predictive model of AKI progression to CKD included externally validated study also showed that higher discharge Scr was an independent risk factor for progression to advanced CKD in AKI patients (OR = 37.01, 95% CI 19.46–70.37) [20].

Although AKI is generally reversible, some patients may experience incomplete recovery of renal function, while others experience subsequent accelerated renal loss leading to an increased risk of CKD [34]. In a 10-year study of 3679 patients, AKI episode was an important risk factor for the progression of renal function to stage 4 CKD (HR = 3.56, 95% CI 2.76–4.71), and each episode of AKI doubled that risk (HR = 2.02, 95% CI 1.78–2.30) [35]. Our study showed that the 90-day renal function failure rate was 66.8%, while a retrospective cohort study showed that the 7-day failure rate of renal function recovery in diabetes patients with AKI was about 46.9% [36]. It can be seen that the short-term renal prognosis of diabetic patients with AKI is poor. However, the differences in renal function recovery rates between previous studies and this study may be related to differences in basic characteristics of patients, disease severity, follow-up time and treatment strategies [37].

Incomplete recovery from AKI can lead to chronic dysfunction and progressive decline in renal function, especially in patients with pre-existing CKD [38]. Poorly healed of tubules have been reported to cause disproportionate scarring, leading to loss of peritubular capillaries, resulting in volume-dependent salt-sensitive hypertension and subsequent glomerular damage [39]. Renal damage affecting structure and function could be classified as acute or chronic at 3 months. Clinical practice guidelines also recommend a 3-month follow-up to assess whether patients with AKI develop CKD [40, 41]. Therefore, 90 days after the occurrence of AKI was selected as the time cutoff to observe the recovery of renal function in this study, and a clinical prediction model for short-term non-recovery of renal function in diabetic patients with AKI was developed.

However, this study has several limitations. First, this is a single-center retrospective study with limited sample size and rather short-term following up. Second, the diagnosis of AKI was only based on changes in Scr, and only cases of AKI with decreased urine output might be missed. Third, Scr values were only collected at baseline and 90 days after AKI, which lacked dynamic variation values. Therefore, it is not possible to determine whether patients develop AKI more than once. Fourth, this study only carried out internal verification and did not include external verification. It is still necessary to further expand the sample size for external verification of the predictive performance of the model.

## Conclusion

Increase of diabetes duration, history of hypertension, CKD, decrease of PLT, 25(OH)D3, increase of PBG and discharged Scr are independent risk factors for non-recovery of renal function in diabetic patients with AKI within 90 days. Based on the above variables, a nomogram can be used to establish a prediction model to help predict the short-term renal prognosis, which has been verified to have good discrimination, calibration and good clinical practicability.

## Data Availability

Considering the privacy of patients, if readers have similar research and want to obtain data related to the article, they can contact the corresponding author, the corresponding research data can be obtained with permission.
